# 被膜金属支架在气道瘘治疗中的应用研究

**DOI:** 10.3779/j.issn.1009-3419.2011.08.08

**Published:** 2011-08-20

**Authors:** 洪武 王, 冬妹 李, 楠 张, 珩 邹, 凌飞 罗, 洪明 马, 云芝 周, 晶 李, 素娟 梁

**Affiliations:** 100028 北京，煤炭总医院肿瘤微创治疗中心 Minimal Invasive Tumor Therapy Center, Meitan General Hospital, Beijing 100028, China

**Keywords:** 肺肿瘤, 食管气管瘘, 支气管胸膜瘘, 金属支架, Lung neoplasms, Esophageorespiratory fistula, Bronchopleural fistula, Stents

## Abstract

**背景与目的:**

气道瘘包括食管气管瘘、支气管胸膜瘘和气管纵隔瘘，临床治疗较为棘手。本文旨在探讨被膜金属支架封堵气道瘘的疗效和安全性。

**方法:**

回顾性分析32例食管气管瘘、5例支气管胸膜瘘和1例气管纵隔瘘患者在气管镜和/或X线透视下放置被膜金属支架。原发病为食管肿瘤26例，肺癌11例，甲状腺癌1例。

**结果:**

38例患者共有46个瘘口，口径0.5 cm-7.0 cm。放置Z型气管被膜金属支架40枚(其中Y形24枚，L形8枚和I形8枚)。食管放置被膜金属支架24枚。46个瘘口封堵疗效：治愈2例(4.3%)，临床完全缓解28例(60.9%)，部分缓解11例(23.9%)，无效5例(10.9%)，有效率为89.1%，中位生存时间为5个月。

**结论:**

被膜金属支架能有效封堵食管气管瘘、支气管胸膜瘘和气管纵隔瘘。应首选气管支架封堵ERF，无效的患者可同时放置食管支架。分叉型气管支架尤其适于隆突周围瘘口。

## Abstract (en)

**Background and objective:**

Treating airway fistulas, including esophagorespiratory fistulas (ERFs), bronchopleural fistulas (BPFs), and tracheomediastinal fistulas (TMFs), is difficult. The aim of this study is to evaluate the safety and clinical efficacy of metallic covered Z-type stents (CZTS) for the treatment of airway fistulas through bronchoscopy or fluroscopy.

**Methods:**

Thirty-eight patients with fistulas between the esophagus, mediastina, and airways (32 ERFs, 5 BPFs, and 1 TMF) were retrospectively reviewed after treatment with covered metallic esophageal and airway stents. The fistulas were caused by esophageal (*n*=26), bronchogenic (*n*=11), and thyroid (*n*=1) carcinomas.

**Results:**

Forty-six fistulas were found in 38 patients. The fistula size ranged from 0.5 cm to 7.0 cm. Forty airway covered metal stents (24 Y-type, 8 L-type, and 8 Ⅰ-type) and 24 esophageal metal stents were placed. Complete responses to the sealing effects of fistulas were noted in 4.3% of all the fistulas, 60.9% showed complete clinical responses, 23.9% showed partial responses, and 10.9% showed no response. An effectivity rate of 89.1% was observed, and the median survival duration of all patients was 5 months.

**Conclusions:**

The use of CZTS appears to be safe and feasible for the palliative treatment of ERFs, BPFs, and TMFs. Airway stent placement is recommended for patients with ERF. In the event that airway stents fail, esophageal stents should be given. Airway bifurcation stents were observed to be especially suitable for the sealing of fistulas near the trachea carina.

气道瘘包括食管气管瘘(esophagorespiratory fistula, ERF)、支气管胸膜瘘(broncho-pleural fistula, BPF)和气管纵隔瘘(tracheo-mediastinal fistula, TMF)。瘘口位于气管或支气管，可通向食管、胃、纵隔或胸腔等，临床治疗极为棘手。近年来由于气管支架和食管支架的广泛应用，大多数患者取得较好疗效^[1-3]^。ERF一般通过放置食管支架来解决^[3]^，而BPF和TMF只能通过放置气管支架来解决。但对不能放置食管支架或放置食管支架失败的患者，放置气管支架亦可取得较好疗效^[4, 5]^。近年来作者在采用被膜金属支架堵瘘的治疗中取得一定经验，供临床借鉴。

## 1 资料与方法

### 1.1 病例资料

2006年9月-2011年1月北京煤炭总医院共收治32例ERF、5例BPF和1例TMF的患者，男24例，女14例；年龄46岁-76岁，平均(58.2±1.4)岁。原发病包括食管疾病26例(24例为食管癌，2例为食管非霍杰金淋巴瘤)，肺癌11例，甲状腺癌1例。研究方案经煤炭总医院伦理委员会批准，受试对象均签署了书面知情同意书。

### 1.2 支架材料

均应用可回收Z型被膜气管支架(CZTS) (图 1，江苏西格玛医用实业有限公司提供)和食管支架。气管支架直管形(I形)8例，Y形24例，L形8例(被膜为硅橡胶制成，内含锯齿形不锈钢丝骨架)。气管支架内径为16 mm，长度为50 mm-70 mm。分叉支架(Y形和L形)左主支气管支架内径为12 mm-13 mm，长度为30 mm-45 mm；右主支气管支架内径为13 mm-15 mm，长度为15 mm。根据患者的需要选择合适的长度及管径或特殊定做。食管支架的规格为支架内径为17 mm-18 mm，长度为60 mm-140 mm，食管下端支架为防返流型。

**1:**
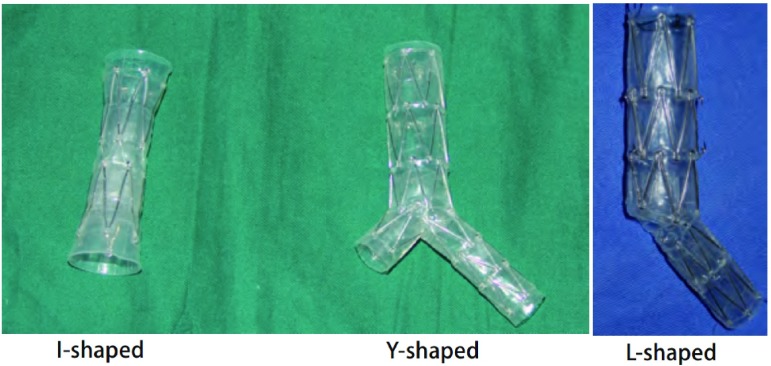
Z型被膜支架 Covered Z-type stents(CZTS)

由外院转来的患者所用支架不统一。食管支架均为双蘑菇头型被膜镍钛记忆合金网状支架(国内多个厂家生产)，规格为17 mm-20 mm/60 mm-140 mm。气管支架亦为镍钛记忆合金网状支架，部分被膜。

### 1.3 适应证

不适合放置食管支架或已放置食管支架失败的患者，尤其对近隆突附近的ERF均适宜放置气管支架。Y形支架主要用于近隆突处ERF或气管上端瘘口较大者；L形支架亦主要用于近隆突处ERF和BPF，或隆突较宽不适宜放置Y形支架者；I形支架主要用于气管中上端ERF、TMF，或用于一侧支气管瘘。

### 1.4 气促症状的评分

按照美国胸科协会的气促评级标准^[6]^进行评级。0级：正常；1级：快步走时出现气促；2级：平常速度步行时出现气促；3级：平常速度步行时因出现气促而停止步行；4级：轻微活动后出现气促。

### 1.5 禁忌证

伴有一侧支气管闭塞或肺不张。

### 1.6 瘘口封堵疗效的判断标准

目前国内外尚无气管瘘疗效判断标准，作者根据自己的经验制定瘘口封堵疗效的判断标准。完全缓解(complete response, CR)：瘘口愈合，临床症状完全缓解持续1个月；临床完全缓解(clinical complete response, cCR)：瘘口未愈合，但被支架完全封堵，临床症状(饮水呛咳、发热等)完全缓解持续1个月；部分缓解(partial response, PR)：瘘口未闭合，部分被支架封堵，临床症状部分缓解；无效(no response, NR)：瘘口未闭合，未被支架封堵，临床症状无缓解。

### 1.7 支架置入方法

I形支架在内镜引导下置入，L形和Y形则在硬镜或内镜和X线双重引导下置入。食管支架主要在X线透视下置入。Z型被膜支架输送器由带有导引头(输送鞘内芯)的支架输送鞘、装有支架的内管和支架后方的顶推管组成，故又称为三套管放置法。

### 1.8 统计处理

采用SPSS 16.0进行数据分析，治疗前后比较采用配对*t*检验，以*P* < 0.05为有统计学差异。

## 2 结果

### 2.1 病因

46个瘘口中癌性17个(37.0%)，良性29个(63.0%)。有些恶性疾病发生的瘘为良性，如2例食管非霍奇金淋巴瘤虽为恶性疾病，但发生的瘘却是术中损伤所致，无肿瘤残留，应为良性瘘。肺癌中有些瘘也是术后发生，如吻合口瘘或放疗所致瘘也是良性。表 1中描述的良、恶性瘘均经严格审核，大多数经病理检查确诊。病因以肿瘤浸润和放射损伤最常见，分别为14例(30.4%)和16例(34.8%)，其它依次为手术中损伤8例(17.4%)、支架损伤4例(8.7%)、支架破裂3例(6.5%)和光动力治疗后1例(2.2%)。4例支架损伤均为食管支架上缘将食管和气管切割所致。3例支架破裂中2例为肺癌患者放疗后发生ERF，放置被膜金属食管支架1年后支架破裂，另1例为食管癌患者放疗后发生ERF，放置被膜金属气管支架1年后支架破裂。多发瘘口和发生瘘口较大者均为放疗后患者。瘘口最小0.5 cm×0.5 cm，最大为7.0 cm×3.0 cm，平均(1.8±0.3)cm。

**1:** 瘘口发生的部位及病因 The site and cause of fistula

Fistula site	*n*	Causes of fistula					
		Tumor infiltration	Radiation injury	Intraoperative injury	Stent injury	Stent fracture	Photodynamic therapy
Trachea							
Upper 1/3	3	0	1	0	1	1	0
Middle 1/3	6	4	0	0	2	0	0
Lower 1/3	12	1	4	3	1	2	1
Middle & lower 2/3	5	4	1	0	0	0	0
Right bronchus (RB)							
Surgical stump	3	1	0	2	0	0	0
Orifice of RB	7	1	3	3	0	0	0
Orifice of right middle and lower lobe	2	0	2	0	0	0	0
Left bronchus							
Surgical stump	2	1	1	0	0	0	0
Left main bronchus	6	2	4	0	0	0	0
Total	46	14	16	8	4	3	1

### 2.2 部位

由表 1可见瘘口主要位于主气管26个(其中以中下段为主)，占56.5%；右侧支气管12个(26.1%)，左侧支气管8个(17.4%)，其中同时累及主气管和双侧支气管4例，不同部位2个以上瘘口4例(其中1例同时有3个瘘口)，BPF 5例，TMF 1例。一个患者可有多个瘘口，因此瘘口的数量多于患者数量。

### 2.3 就诊时间

从发生瘘到来北京煤炭总医院就诊最短1天，最长5个月，平均(37.5±6.2)天。36例ERF患者均有明显的进食和饮水呛咳。所有患者均已禁食，最长5个月，明显消瘦甚至恶液质，并伴有不同程度的肺部感染和呼吸困难。5例BPF的患者均伴有患侧脓胸。

### 2.4 支架类型

放置Z型气管被膜金属支架40枚，其中Y形24枚、L形8枚和I形8枚(图 1，图 2)。3例多发ERF的患者分别放置了2个气管支架。食管放置被膜金属支架24枚。食管和气管同时放置支架21例(其中1例食管内放置5枚支架)。18例先放置食管支架失败后又置入气管支架，其中3例因网状食管支架上段切开造成ERF，遂将食管支架取出，1例又置入Z型食管被膜金属支架。3例为气管支架置入后仍有瘘又置入食管支架(图 3A，图 3B)。5例BPF均置入L型支架，1例TMF置入I型支架。

**2:**
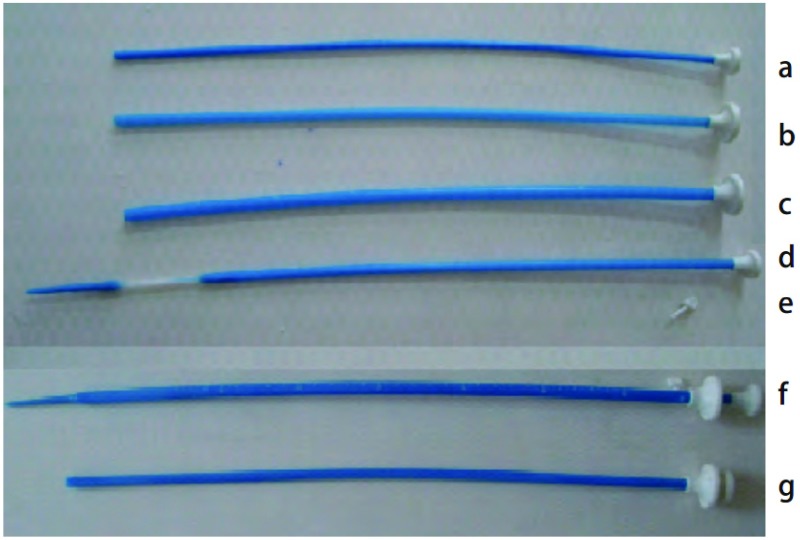
Z型被膜支架输送器。a：顶推管；b：支架内管；c：支架输送鞘；d：导引头(输送鞘内芯)；e：插销；f带有导引头(输送鞘内芯)的支架输送鞘(b+d)；g：装有支架的内管(a+c)。 Covered Z-type stent delivery device. a: Pushing tube; b: Inner tube containing stent; c: Stent delivery sheath; d: Seeker (delivery sheath core); e: Bolt; f: Stent delivery sheath with a seeker (delivery sheath core) (b+d); g: Inner tube containing stent (a+c).

**3:**
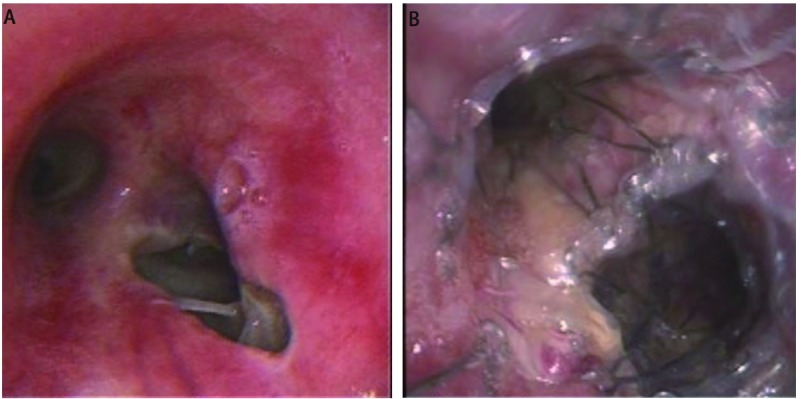
肺癌放疗后ERF(男，53岁)。A：气管下端及右主支气管开口后壁1 cm×1.5 cm缺损；B：气管内放置Y形被膜金属支架(主气管17 mm×50 mm/左主支气管12 mm ×15 mm/右主支气管12 mm×35 mm)，支架在右上叶开口处行破膜处理。本例患者同时放置食管支架。 ERF after radiotherapy in patient with lung cancer (male, 53 years old). A: Tracheobronchialesophagus fistula (1 cm×1.5 cm) located around carina; B: Y-shaped CZTS in airway (main bronchus 17 mm×50 mm / left main bronchus 12 mm× 15 mm/right main bronchus 12 mm×35 mm), and the cover was opened in the orifice of right upper bronchus.

### 2.5 瘘口封堵的疗效

由表 2可见，4.3%的患者CR，2例均为术中损伤所致，瘘口4月和1年后完全愈合，支架取出。临床症状完全缓解率(CR+cCR)为65.1%，临床获益率(CR+cCR+PR)为89.1%。5例NR的患者主要是因为瘘口位于主气管中、上段和瘘口巨大(> 3 cm)。2例右下ERF分别放置食管支架达cCR，因瘘口位于下叶支气管开口无法放置支气管支架。2例气管腺样囊性癌的患者在气管中下段发生巨大ERF(主气管瘘口长径分别达6 cm和7 cm)，放置Y形气管支架联合食管支架后达cCR。5例BPF(图 4A，图 4B)和1例TMF均达cCR。

**4:**
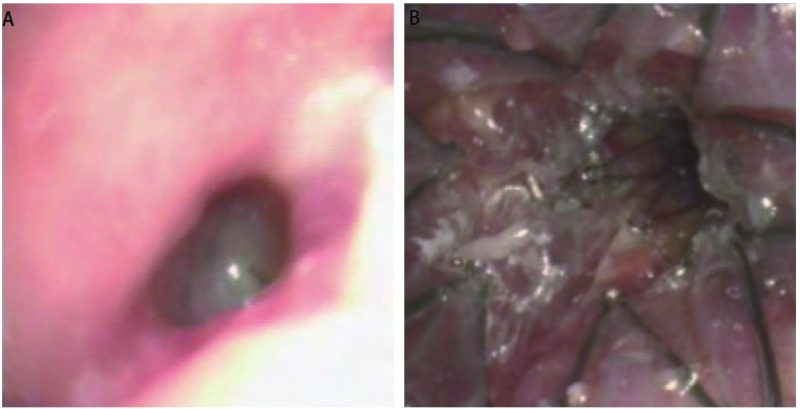
肺癌左全肺切除术后支气管胸膜瘘(男，49岁)。A：左主支气管残端可见瘘口(0.3 cm×0.5 cm)，可见大量白色分泌物涌出，瘘口周围可见复发的肿瘤；B：气管及右主支气管内放置L形被膜金属支架(主气管17 mm× 60 mm/右主支气管13 mm×35 mm)，支架左侧开口封闭。 BPF of the left main bronchus in patient with lung cancer after left pneumonectomy (male, 49 years old). A: Stump fistula of the left main bronchus (0.3 cm×0.5 cm fistua) in patient with lung cancer after left pneumonectomy; B: L-shaped CZTS (left orifice of the stent was sealed) was placed in trachea and right bronchus (Trachea 17 mm×60 mm/Right bronchus 13 mm×35 mm).

**2:** 支架封堵瘘口的效果 Sealing efficiency of fistulas with stents

Fistula site	*n*	CR	cCR	PR	NR
Trachea					
Upper 1/3	3	0	0	1	2
Middle 1/3	6	0	4	1	1
Lower 1/3	12	2	10	0	0
Middle & lower 2/3	5	0	2	2	1
Right bronchus(RB)					
Surgical stump	3	0	2	0	1
Orifice of RB	7	0	2	5	0
Orifice of right middle and lower lobe	2	0	2	0	0
Left bronchus					
Surgical stump	2	0	1	1	0
Left main bronchus	6	0	5	1	0
Total	46	2	28	11	5
CR: complete respone; cCR: clinical complete response; PR: partial response; NR: no response.					

38例患者体质评分(karnofsky physical score, KPS)术前、术后分别为44.2±2.7和70.4±3.2(*t*=6.26, *P* < 0.001)，气促评分术前、术后分别为3.1±0.1和1.4±0.2(*t*=7.60, *P* < 0.001)。

### 2.6 并发症

42例次(其中4例两次置入支架)支架置入术后1周内痰液粘附在支架上，经气管镜吸痰及加强雾化处理后痰液渐减少。19例(45.2%)支架置入1个月后支架两端有肉芽组织形成，经氩等离子体凝固(argon plasma coagulation, APC)及CO_2_冷冻处理后肉芽减少，未造成气管堵塞。30例次(71.4%)置入支架后引起短暂胸痛，只有2例因胸痛难忍将支架取出(均同时放置食管支架)，其余经1周左右的对症处理渐缓解。1例(2.4%)L型支架置入后咳嗽剧烈，2天后支架移位(全部进入气管)，将支架取出后重新置入，未再发生移位。

### 2.7 随访

36例患者随访1个月-3年。中位生存时间为5个月，失访2例，最长1例存活时间已逾3年(痊愈)。4例(4/21, 19%)同时放置气管和食管支架的患者因大咯血死亡，发生时间为术后2天、1周、5月和10月。6例(15.8%)因全身衰竭死亡，1例因并发化学肺炎/呼吸衰竭死亡。

## 3 讨论

ERF根据病因可分为良性和恶性，由甲状腺、纵隔淋巴及食管的恶性肿瘤引起者约占50% - 6 0%，其它则来自创伤、炎症或原因不明^[3]^。癌组织侵犯食管和气管后癌组织坏死可形成ER F及食管纵隔瘘，放疗杀死肿瘤组织的同时损伤正常组织也可形成瘘，放疗和化疗后肿瘤急剧坏死而正常组织修复障碍亦可形成瘘。瘘口大、瘘周组织炎症、瘘区正常组织修复障碍和进食障碍使全身营养不良，导致瘘长期不愈。本组气道瘘的病因中食管肿瘤26例(68.4%)，肺癌11例(28.9%)，甲状腺癌1例(2.6%)。值得注意的是46个瘘口中癌浸润和放疗后所致占65.2%，部分患者两种因素都有，26个源于食管癌的瘘口术中气管损伤和食管支架损伤两种人为因素所致12个(46.1%)。

ERF的诊断不难，临床上出现饮水呛咳、肺部感染等应考虑诊断，通过气管镜、胃镜及食管造影等可确诊。危害主要是难以控制的肺部感染和由于禁食所致的营养不良。BPF、TMF主要根据手术史、通过气管镜检查及气管造影可确诊，危害主要是难以控制的胸腔感染。

气管瘘的封堵中裸支架无法封堵瘘口，只有通过被膜金属支架或硅酮支架方能奏效。选择放置气管支架还是食管支架应根据患者病情而定。一般认为由食管癌引起的ERF伴有食管狭窄而无或轻度气管狭窄时，带膜食管支架应放于食管内，效果立竿见影^[2-4]^。但由于食管支架易移位，所以术后易复发。当狭窄位于气管时，则支架应置入气管内。当食管和气管均有中至重度狭窄或瘘口较大时，则需在两侧同时放置支架。分叉支架可有效封堵瘘口，同时也能有效封堵两侧支气管，进一步防止异物从瘘口内进入气管，起到双保险的作用，而且分叉气管支架固定良好，一般不易移位，并发症较少，对不适合食管支架的患者可作为首选^[7]^。有些情况不适宜或无法应用食管支架，如食管癌已经手术切除(胸腔胃、结肠代食管或食管缩短)、已放置食管支架而出现气管狭窄或其它原因不适合食管支架置入的ERF患者，用带膜气管支架治疗，也取得非常好的疗效^[5]^。

本组24枚Y形支架主要用于主气管下端或支气管-食管瘘，或气管上端瘘口较大者。8枚L形支架主要用于支气管残端-胸膜瘘和近隆突处ERF，或隆突较宽不适宜放置Y形支架者；I形支架主要用于中上端ERF，或用于一侧支气管瘘。

目前尚无判断气道瘘疗效的标准。作者根据自己的临床经验初定判断标准供临床参考。有些良性病变，经内支架等治疗后，瘘口可完全愈合，但时间尚难断定。本组CR 4.3%，2例均为术中损伤所致。有些患者虽然瘘口组织上未愈合，但由于内支架的有效封堵，饮水呛咳、发热等症状可完全缓解，可正常进食，KPS等情况明显改善。本组约2/3的患者临床症状完全缓解(CR+cCR)，临床获益率为89.1%，5例NR的患者主要是因瘘口位于气管上段、右主支气管开口或右中间段支气管下端，支架难以封堵严密。18例先放置食管支架失败又置入气管支架后，13例达cCR，其中4例因放置蘑菇头型网状食管支架切割损伤引起瘘口，3例将6个食管支架取出(1例有5个支架)，1例未取，又分别放置Y形气管支架均达cCR。本组有2例瘘口发生于右下叶开口和右下叶背段开口，难以放置支气管支架，只能通过放置食管支架来治疗，取得满意疗效。本组1例气管上端瘘口用直筒型Z型支架未能奏效，主要与瘘口较大及支架密封不严有关，1例放置镍钛记忆合金自膨胀气管支架效果较好，支架置入后可以贴壁紧密，瘘口封堵完全。

一侧肺全切由手术残端引起的支气管胸膜瘘的治疗主要是置入患侧封闭的L形支架。支气管内的支架要适当长些，以充分阻挡瘘口内的分泌物流入健侧肺内。但右侧支气管内的支架要将右上叶开口的膜剪开，以便于通气。

气管和食管两侧同时放置支架时，可使瘘口逐渐扩大，严重时可伴发大出血而死亡。本组4例发生大出血，发生率(19.0%)，应予重视，特别是瘘口较大的患者更易发生大咯血。支架置入前如果肿瘤表面有破溃或肿瘤突出黏膜，可先用APC将肿瘤烧灼，以便于止血和消融肿瘤，然后择时置入支架。

ERF的患者均伴有严重呼吸道感染，气管内分泌物较多，支架置入后易出现痰液滞留^[8]^。支架置入后第3天、第7天应及时行气管镜检，将分泌物吸出，同时加强全身抗感染及营养支持治疗。经气管镜反复处理及雾化处理后，痰液一般不会再增多，也不会引起严重的气管阻塞，这就是气管镜的优势。

支架置入后有一过性胸痛和不适，经对症处理1周后可好转。1例患者置入L型支架后咳嗽剧烈，支架移位，将支架取出后重新放置，并给予止咳、雾化等处理，未再发生移位。

支架置入对封堵瘘口快速、有效，可迅速改善患者的症状，延长患者生命，本组患者置入支架后大多数KPS和气促评分均有明显好转，中位生存时间5个月，长于文献报道的2.8个月和65.5天^[2, 3]^。虽然本组多数为恶性肿瘤患者，但瘘口有良、恶性之分。有6例晚期患者置入后很快死于恶液质和呼吸衰竭，因此，早期治疗非常重要。
